# Beyond the Global Brain Differences: Intra-individual Variability Differences in 1q21.1 Distal and 15q11.2 BP1-BP2 Deletion Carriers

**DOI:** 10.1016/j.biopsych.2023.08.018

**Published:** 2023-09-01

**Authors:** Rune Boen, Tobias Kaufmann, Dennis van der Meer, Oleksandr Frei, Ingrid Agartz, David Ames, Micael Andersson, Nicola J. Armstrong, Eric Artiges, Joshua R. Atkins, Jochen Bauer, Francesco Benedetti, Dorret I. Boomsma, Henry Brodaty, Katharina Brosch, Randy L. Buckner, Murray J. Cairns, Vince Calhoun, Svenja Caspers, Sven Cichon, Aiden P. Corvin, Benedicto Crespo Facorro, Udo Dannlowski, Friederike S. David, Eco J.C. de Geus, Greig I. de Zubicaray, Sylvane Desrivières, Joanne L. Doherty, Gary Donohoe, Stefan Ehrlich, Else Eising, Thomas Espeseth, Simon E. Fisher, Andreas J. Forstner, Lidia Fortaner Uyà, Vincent Frouin, Masaki Fukunaga, Tian Ge, David C. Glahn, Janik Goltermann, Hans J. Grabe, Melissa J. Green, Nynke A. Groenewold, Dominik Grotegerd, Tim Hahn, Ryota Hashimoto, Jayne Y. Hehir-Kwa, Frans A. Henskens, Avram J. Holmes, Asta K. Håberg, Jan Haavik, Sebastien Jacquemont, Andreas Jansen, Christiane Jockwitz, Erik G. Jönsson, Masataka Kikuchi, Tilo Kircher, Kuldeep Kumar, Stephanie Le Hellard, Costin Leu, David E. Linden, Jingyu Liu, Robert Loughnan, Karen A. Mather, Katie L. McMahon, Allan F. McRae, Sarah E. Medland, Susanne Meinert, Clara A. Moreau, Derek W. Morris, Bryan J. Mowry, Thomas W. Mühleisen, Igor Nenadić, Markus M. Nöthen, Lars Nyberg, Michael J. Owen, Marco Paolini, Tomas Paus, Zdenka Pausova, Karin Persson, Yann Quidé, Tiago Reis Marques, Perminder S. Sachdev, Sigrid B. Sando, Ulrich Schall, Rodney J. Scott, Geir Selbæk, Elena Shumskaya, Ana I. Silva, Sanjay M. Sisodiya, Frederike Stein, Dan J. Stein, Benjamin Straube, Fabian Streit, Lachlan T. Strike, Alexander Teumer, Lea Teutenberg, Anbupalam Thalamuthu, Paul A Tooney, Diana Tordesillas-Gutierrez, Julian N Trollor, Dennis van 't Ent, Marianne B.M. van den Bree, Neeltje E.M. van Haren, Javier Vázquez-Bourgon, Henry Völzke, Wei Wen, Katharina Wittfeld, Christopher R.K. Ching, Lars T. Westlye, Paul M. Thompson, Carrie E. Bearden, Kaja K. Selmer, Dag Alnæs, Ole A. Andreassen, Ida E. Sønderby

**Affiliations:** 1 Department of Medical Genetics, Oslo University Hospital, Oslo, Norway; 2 NORMENT, Division of Mental Health and Addiction, Oslo University Hospital and Institute of Clinical Medicine, University of Oslo, Oslo, Norway; 3 Department of Psychiatry and Psychotherapy, Tübingen Center for Mental Health, University of Tübingen, Germany; 4 School of Mental Health and Neuroscience, Faculty of Health, Medicine and Life Sciences, Maastricht University, Maastricht, Netherlands; 5 Centre for Bioinformatics, Department of Informatics, University of Oslo, Oslo, Norway; 6 NORMENT, Institute of Clinical Medicine, University of Oslo, Oslo, Norway; 7 Department of Clinical Research, Diakonhjemmet Hospital, Oslo, Norway; 8 Centre for Psychiatry Research, Department of Clinical Neuroscience, Karolinska Institutet & Stockholm Health Care Services, Stockholm, Sweden; 9 University of Melbourne Academic Unit for Psychiatry of Old Age, St George's Hospital, Kew, VIC, Australia; 10 National Ageing Research Institute, Parkville, VIC, Australia; 11 Department of Integrative Medical Biology (IMB), Umeå University, Umeå, Sweden; 12 Umeå Center for Functional Brain Imaging, Umeå University, Umeå, Sweden; 13 Mathematics & Statistics, Curtin University, Perth, WA, Australia; 14 INSERM U1299, ENS Paris Saclay, Université Paris Saclay, Gif-sur-Yvette, France; 15 EPS Barthelemy Durand, Etampes, France; 16 School of Biomedical Sciences and Pharmacy, University of Newcastle, Callaghan, NSW, Australia; 17 Precision Medicine Research Program, Hunter Medical Research Institute, Newcastle, NSW, Australia; 18 The Cancer Epidemiology Unit, Nuffield Department of Population Health, University of Oxford, Oxford, UK; 19 University Clinic for Radiology, University of Münster, Muenster, Germany; 20 University Vita-Salute San Raffaele, Milano, Italy; 21 Psychiatry & Clinical Psychobiology, Division of Neuroscience, Scientific Institute Ospedale San Raffaele, Milano, Italy; 22 Department of Biological Psychology, Vrije Universiteit Amsterdam, Amsterdam, NH, Netherlands; 23 Centre for Healthy Brain Ageing, University of New South Wales, Sydney, NSW, Australia; 24 Department of Psychiatry and Psychotherapy, Philipps-University Marburg, Marburg, Germany; 25 Psychology and Center for Brain Science. Harvard University, Cambridge, MA, USA; 26 Psychiatry, Massachusetts General Hospital, Boston, MA, USA; 27 Precision Medicine Research Program, Hunter Medical Research Institute, New Lambton Heights, NSW, Australia; 28 Tri-institutional Center for Translational Research in Neuroimaging and Data Science (TReNDS), Georgia State, Georgia Tech, Emory, Atlanta, GA 30303, USA; 29 Institute of Neuroscience and Medicine (INM-1), Research Centre Jülich, Jülich, Germany; 30 Institute for Anatomy I, Medical Faculty & University Hospital Düsseldorf, Heinrich Heine University Düsseldorf, Düsseldorf, Germany; 31 University of Basel, Basel, Switzerland; 32 University Hospital Basel, Basel, Switzerland; 33 Department of Psychiatry, Trinity College Dublin, Dublin, Ireland; 34 Hospital Universitario Virgen del Rocío/ IBIS/ CSICC, Sevilla, Spain; 35 Centro de Investigación Biomedica en Red Salud Mental (CIBERSAM), Sevilla, Spain; 36 Department of Psychiatry, University of Sevilla, Sevilla, Spain; 37 Institute for Translational Psychiatry, University of Münster, Münster, Germany; 38 Institute of Human Genetics, University of Bonn, School of Medicine & University Hospital Bonn, Bonn, Germany; 39 School of Psychology and Counselling, Queensland University of Technology, Brisbane, QLD, Australia; 40 Social Genetic and Developmental Psychiatry Centre, Institute of Psychiatry, Psychology & Neuroscience, King's College London, London, UK; 41 Centre for Neuropsychiatric Genetics and Genomics, Cardiff University, Cardiff, UK; 42 Cardiff University’s Brain Research Imaging Centre, School of Psychology, Cardiff University, Cardiff, UK; 43 School of Psychology & Center for Neuroimaging, Cognition and Genomics, University of Galway, Galway, Ireland; 44 Translational Developmental Neuroscience Section, Division of Psychological and Social Medicine and Developmental Neurosciences, Faculty of Medicine, TU Dresden, Germany; 45 Language and Genetics Department, Max Planck Institute for Psycholinguistics, Nijmegen, Netherlands; 46 Department of Psychology, University of Oslo, Oslo, Norway; 47 Department of Psychology, Oslo New University College, Oslo, Norway; 48 Donders Institute for Brain, Cognition and Behaviour, Radboud University, Nijmegen, Netherlands; 49 Université Paris-Saclay, Neurospin, CEA, Gif sur Yvette, France; 50 Division of Cerebral Integration, National Institute for Physiological Sciences, Okazaki, Japan; 51 Psychiatric and Neurodevelopmental Genetics Unit, Center for Genomic Medicine, Massachusetts General Hospital, Boston, MA, USA; 52 Department of Psychiatry, Massachusetts General Hospital, Harvard Medical School, Boston, MA, USA; 53 Department of Psychiatry and Behavioral Sciences, Boston Children's Hospital, Boston, MA, USA; 54 Department of Psychiatry, Harvard Medical School, Boston, MA, USA; 55 Department of Psychiatry and Psychotherapy, University Medicine Greifswald, Greifswald, Germany; 56 Discipline of Psychiatry and Mental Health, School of Clinical Medicine, University of New South Wales, Sydney, NSW, Australia; 57 Neuroscience Research Australia, Sydney, NSW, Australia; 58 Department of Psychiatry & Mental Health, Neuroscience Institute, University of Cape Town, Cape Town, South Africa; 59 Department of Pathology of Mental Diseases, National Institute of Mental Health, National Center of Neurology and Psychiatry, Kodaira, Tokyo, Japan; 60 Princess Máxima Center for Pediatric Oncology, Utrecht, Netherlands; 61 School of Medicine and Public Health, University of Newcastle, Newcastle, NSW, Australia; 62 PRC for Health Behaviour, University of Newcastle, Newcastle, NSW, Australia; 63 Psychiatry, Rutgers University, New Brunswick, NJ, USA; 64 Brain Health Institute, Rutgers University, Piscataway, NJ, USA; 65 Department of Neuromedicine and Movement Science (INB), Faculty of Medicine and Health Sciences, NTNU, Trondheim, Norway; 66 Department of Radiology and Nuclear Medicine, St. Olav's Hospital, Trondheim, Norway; 67 Department of Biomedicine, University of Bergen, Bergen, Norway; 68 Division of Psychiatry, Haukeland University Hospital, Bergen, Norway; 69 Sainte Justine Hospital Research Center, Montreal, QC, Canada; 70 Department of Pediatrics, University of Montreal, QC, Canada; 71 Core-Facility Brainimaging, Marburg, Germany; 72 Department of Psychiatry, Marburg, Germany; 73 Centre for Psychiatry Research, Department of Clinical Neuroscience, Karolinska Institutet & Stockholm Health Care Services, Stockholm Region, Stockholm, Sweden; 74 Department of Genome Informatics, Graduate School of Medicine, Osaka University, Osaka, Japan; 75 Department of Computational Biology and Medical Sciences, Graduate School of Frontier Science, The University of Tokyo, Chiba, Japan; 76 Centre de recherche CHU Sainte-Justine and University of Montréal, Montral, QC, Canada; 77 NORMENT, Department of Clinical Science, University of Bergen, Bergen, Norway; 78 Dr. Einar Martens Research Group for Biological Psychiatry, Center for Medical Genetics and Molecular Medicine, Haukeland University Hospital, Bergen, Norway; 79 Department of Clinical and Experimental Epilepsy, Institute of Neurology, University College London, London, UK; 80 Genomic Medicine Institute, Lerner Research Institute, Cleveland Clinic, Cleveland, OH, USA; 81 Neuroscience and Mental Health Innovation Institute, Cardiff University, Cardiff, UK; 82 School for Mental Health and Neuroscience, Department of Psychiatry and Neuropsychology, Faculty of Health, Medicine and Life Sciences, Maastricht University, Maastricht, Netherlands; 83 Department of Computer Science, Georgia State University, GA, USA; 84 Center for Translational Research in Neuroimaging and Data Science, Georgia State University, Atlanta, GA, USA; 85 Department of Cognitive Science, University of California San Diego, La Jolla, CA, USA; 86 Population Neuroscience and Genetics, University of California San Diego, La Jolla, CA, USA; 87 School of Clinical Sciences, Queensland University of Technology, Brisbane, QLD, Australia; 88 Institute for Molecular Bioscience, The University of Queensland, Brisbane, Australia; 89 Psychiatric Genetics, QIMR Berghofer Medical Research Institute, Brisbane, QLD, Australia; 90 University of Queensland, Brisbane, QLD, Australia; 91 Queensland University of Technology, Brisbane, QLD, Australia; 92 Imaging Genetics Center, Mark and Mary Stevens Institute for Neuroimaging and Informatics, Keck School of Medicine, University of Southern California, Marina del Rey, CA, USA; 93 Centre for Neuroimaging, Cognition and Genomics, School of Biological and Chemical Sciences, University of Galway, Galway, Ireland; 94 Queensland Brain Institute, The University of Queensland, Brisbane, QLD, Australia; 95 Queensland Centre for Mental Health Research, The University of Queensland, QLD, Australia; 96 Department of Biomedicine, University of Basel, Basel, Switzerland; 97 Department of Radiation Sciences and Integrative Medical Biology, Umeå University, Umeå, Sweden; 98 Division of Psychological Medicine and Clinical Neurosciences, Cardiff University, Cardiff, UK; 99 Departments of Psychiatry and Neuroscience, Faculty of Medicine and Centre Hospitalier Universitaire Sainte-Justine, University of Montreal, Montreal, QC, Canada; 100 Departments of Psychiatry and Psychology, University of Toronto, ONT, Canada; 101 The Hospital for Sick Children, Toronto, ON, Canada; 102 Department of Physiology, University of Toronto, Toronto, ON, Canada; 103 The Norwegian National Centre for Ageing and Health, Dept. of Geriatric Medicine, Vestfold Hospital Trust, Tønsberg, Norway; 104 Department of Geriatric Medicine, Oslo University Hospital, Oslo, Norway; 105 School of Psychology, The University of New south Wales, Sydney, NSW, Australia; 106 Neuroscience Research Australia, Randwick, NSW, Australia; 107 Psychosis Studies, Institute of Psychiatry, Psychology and Neuroscience, King's College London, London, UK; 108 Neuropsychiatric Insitute, The Prince of Wales Hospital,Sydney, NSW, Australia; 109 Department of Neurology and Clinical Neurophysiology, University Hospital of Trondheim, Trondheim, Norway; 110 Hunter Medical Research Institute, Newqcastle, NSW, Australia; 111 School of Biomedical Sciences and Pharmacy, College of Medicine, Health and Wellbeing, University of Newcastle, Newcastle, NSW, Australia; 112 Division of Molecular Medicine, NSW Health Pathology, Newcastle, NSW, Australia; 113 Level 3 West, Hunter Medical Research Institute, Newcastle, NSW, Australia; 114 Norwegian Centre for Ageing and Health, Vestfold Hospital Trust, Tønsberg, Norway; 115 Faculty of Medicine, University of Oslo, Oslo, Norway; 116 Department of Human Genetics, Radboud University Medical Center, Nijmegen, Netherlands; 117 Chalfont Centre for Epilepsy, Chalfont St Peter, UK; 118 SAMRC Unit on Risk & Resilience in Mental Disorders, Dept of Psychiatry & Neuroscience Institute, University of Cape Town, Cape Town, South Africa; 119 Department of Genetic Epidemiology in Psychiatry, Central Institute of Mental Health, Medical Faculty Mannheim, University of Heidelberg, Mannheim, Germany; 120 School of Psychology and Counselling, Faculty of Health, Queensland University of Technology, Brisbane, Australia; 121 Institute for Community Medicine, University Medicine Greifswald, Greifswald, Germany; 122 German Centre for Cardiovascular Research (DZHK), Greifswald, Germany; 123 Hunter Medical Research Institute, New Lambtion Heights, NSW, Australia; 124 Instituto de Física de Cantabria UC-CSIC, Santander, Spain; 125 Department of Radiology, Marqués de Valdecilla University Hospital, Valdecilla Biomedical Research Institute IDIVAL, Santander, Spain; 126 Department of Developmental Disability Neuropsychiatry, University of New South Wales, Sydney, NSW, Australia; 127 Centre for Healthy Brain Ageing, University of New South Newcastle, NSW, Australia; 128 Biological Psychology & Netherlands Twin Register, Vrije Universiteit Amsterdam, NH, Netherlands; 129 Institute of Psychological Medicine and Clinical Neurosciences, Cardiff, UK; 130 Centre for Neuropsychiatric Genetics and Genomics, Cardiff, UK; 131 Erasmus Medical Centre - Sophie, Rotterdam, Netherlands; 132 University Medical Centre Utrecht, Utrecht, Netherlands; 133 Department of Psychiatry, University Hospital Maqués de Valdecilla - IDIVAL, Santander, Spain; 134 Departamento de Medicina y Psiquiatría, Universidad de Cantabria, Santander, Spain; 135 University Medicine Greifswald, Institute for Community Medicine, Greifswald, Germany; 136 KG Jebsen Centre for Neurodevelopmental Disorders, University of Oslo, Oslo, Norway; 137 Semel Institute for Neuroscience and Human Behavior, Departments of Psychiatry and Biobehavioral Sciences and Psychology, University of California Los Angeles, Los Angeles, CA, USA; 138 Department of Research and Innovation, Division of Clinical Neuroscience, Oslo University Hospital and the University of Oslo, Norway; 139 Kristiania University College, Oslo, Norway

**Keywords:** copy number variants, 1q21.1 distal, 15q11.2 BP1-BP2, intra-individual variability, magnetic resonance imaging, brain structure

## Abstract

**Background:**

The 1q21.1 distal and 15q11.2 BP1-BP2 CNVs exhibit regional and global brain differences compared to non-carriers. However, interpreting regional differences is challenging if a global difference drives the regional brain differences. Intra-individual variability measures can be used to test for regional differences beyond global differences in brain structure.

**Methods:**

Magnetic resonance imaging data were used to obtain regional brain values for 1q21.1 distal deletion (n=30) and duplication (n=27), and 15q11.2 BP1-BP2 deletion (n=170) and duplication (n=243) carriers and matched non-carriers (n=2,350). Regional intra-deviation (RID) scores i.e., the standardized difference between an individual’s regional difference and global difference, were used to test for regional differences that diverge from the global difference.

**Results:**

For the 1q21.1 distal deletion carriers, cortical surface area for regions in the medial visual cortex, posterior cingulate and temporal pole differed less, and regions in the prefrontal and superior temporal cortex differed more than the global difference in cortical surface area. For the 15q11.2 BP1-BP2 deletion carriers, cortical thickness in regions in the medial visual cortex, auditory cortex and temporal pole differed less, and the prefrontal and somatosensory cortex differed more than the global difference in cortical thickness.

**Conclusion:**

We find evidence for regional effects beyond differences in global brain measures in 1q21.1 distal and 15q11.2 BP1-BP2 CNVs. The results provide new insight into brain profiling of the 1q21.1 distal and 15q11.2 BP1-BP2 CNVs, with the potential to increase our understanding of mechanisms involved in altered neurodevelopment.

## Introduction

Carriers of certain rare recurrent copy number variants (CNVs) - i.e., deletions or duplications of a segment of the genome - have a higher risk of developing psychiatric and neurodevelopmental disorders, including schizophrenia and autism spectrum disorder^1–5^ Several rare recurrent CNVs have moderate to large effects on structural brain measures derived from magnetic resonance imaging (MRI)^6,7^. The effects of CNVs on brain structure have been suggested to occur primarily during early neurodevelopment^8^, and some rare recurrent CNVs have been associated with altered cellular function, composition and size derived from cortical organoids that models fetal and early neurodevelopment^9–12^. The 1q21.1 distal and 15q11.2 BP1-BP2 deletions are two of the most common recurrent CNVs^1,13,14^. They yield a higher risk of psychiatric and neurodevelopmental disorders^1–5^ and show moderate to large effects on brain structure^15,16^. Thus, studying 1q21.1 distal and 15q11.2 BP1-BP2 deletion carriers offer a promising genetics-first approach to study deviations in neurodevelopment and brain structure, which may underlie the increased risk of developing psychiatric and neurodevelopmental disorders^5,8^.

To date, the neuroimaging studies on CNVs have focused on conventional mean comparisons between carriers and non-carriers, which have been informative for brain profiling of CNV carriers. For instance, several CNVs have shown global effects on the brain, as demonstrated by group differences in mean cortical thickness, total cortical surface area and total subcortical volume, in addition to wide-spread regional differences^6,7^. However, brain profiling may be challenging if an overall global difference on the brain drives many of the regional mean differences or if regional differences are driven by distinct subgroups in each comparison, rendering inter-regional brain profiles difficult to interpret. To overcome this challenge, detecting brain regions that diverge from the global difference could benefit from intraindividual variability measures, in which regional values represent its position within an individualized brain profile. Identification of brain regions that diverge from the overall global difference of the CNV may provide valuable insights into the regional penetrance, brain organization and functional consequences in CNV carriers. Indeed, as has been demonstrated in other fields such as cognitive science and neuropsychology, e.g.^17–22^, novel scientific and clinical insights can be achieved by looking beyond mean group differences through investigating intraindividual variability.

Both 1q21.1 distal and 15q11.2 BP1-BP2 deletion carriers exhibit global differences in brain structure, with the former displaying a lower total cortical surface area^15^ and the latter showing a higher mean cortical thickness and lower total cortical surface area^16^. Additionally, these deletions also exhibit regional differences across the cortex^15,16^. However, the regional differences vary across the brain as indicated by variation in effect sizes across brain regions. This could indicate that the carriers of the 1q21.1 distal and 15q11.2 BP1-BP2 deletion exhibit higher variability in brain structure, along with systematic inter-regional differences in brain structure as measured by MRI-derived features.

In both 1q21.1 distal and 15q11.2 BP1-BP2 CNV carriers, the largest regional differences are typically found in frontal regions, associated with higher-cognitive processing. In contrast, the posterior brain regions, associated with primary sensory processing, typically do not show significant differences^15,16^. Insight into variation in brain structure may be useful for understanding differences in brain function as cortical morphology overlaps with the functional hierarchical gradient of the brain^23^. This functional hierarchical gradient reflects a sensorimotor (i.e., involved in unimodal and functional specific processes) to association axis (i.e., involved in higher-order cognitive processes) in the human brain^23–25^, which has been supported by anatomical, functional, and evolutionary data^24^. Thus, a more fine-grained brain profile of the structural differences in 1q21.1 distal and 15q11.2 BP1-BP2 CNV carriers may aid our understanding of their phenotypic profile.

Brain structural differences in 1q21.1 distal and 15q11.2 BP1-BP2 CNV carriers indicate global mean differences (i.e., cortical thickness and cortical surface area), as well as regional group differences in primarily frontal brain regions. The regional group differences indicate that some brain regions are more affected than others. Here, we define more affected brain regions as regions that differ more than the global mean difference, and less affected brain regions as regions that differ less than the global mean difference. To measure this, we use an intraindividual variability measure to detect brain regions that diverge from the global difference, where the regional values represent its position within an individualized brain profile. We expected that anterior regions within the association cortices were more affected, whereas posterior regions within the primary sensorimotor cortices were less affected in carriers of the 1q21.1 distal and 15q11.2 BP1-BP2 CNVs.

## Methods and Materials

### Sample

Individuals carrying a 1q21.1 distal or 15q11.2 CNV and a matched non-carrier group were taken from the ENIGMA-CNV working group core dataset and the UK Biobank across 61 scanner sites. Each CNV carrier was matched with five non-carriers based on age, sex, scanner site and ICV using the MatchIt package in R^26^. This resulted in four subsets (sample characteristics are presented in tables 1 and 2, supplementary note 1).

### MRI-derived features, CNVs and quality control

Neuroimaging data were obtained from the UK Biobank, as described elsewhere^27^, and from the ENIGMA-CNV core dataset. The ENIGMA-CNV neuroimaging measures were collected from several sites (see appendix 1 for details) and analyzed using the standardized ENIGMA protocol (https://enigma.ini.usc.edu/protocols/imaging-protocols/). Details of the quality control of the MR images are provided in supplementary note 2. Briefly, the MRI data from the ENIGMA-CNV working group underwent the ENIGMA cortical quality control procedures (https://enigma.ini.usc.edu/protocols/imaging-protocols/), where the 68 cortical and 14 subcortical regions were extracted using the Desikan-Killiany atlas. For the UK Biobank sample, we used the Euler number as a proxy for image quality^28^ and removed all participants with Euler numbers below minus four standard deviations from downstream analyses (n =437). To account for site effects in the samples, we ran each of the four subsets through ComBat, an instrument for data harmonization^29^. CNV calling in ENIGMA-CNV was based on previous publications^15,16^. For the UK Biobank sample, we identified CNVs based on the returned dataset from Crawford et al.^30^ All participants with a CNV as defined in previous publications^15,16,30^ were removed from downstream analyses, except for the individuals flagged with the 1q21.1 distal or the 15q11.2 BP1-BP2 CNV.

### Derivation of dependent variables

We adjusted for the effect of age, age^2^, sex and ICV on every brain regional value using linear regression across the carriers and the non-carriers. The residualized brain regional values were used to calculate the mean and standard deviation for the non-carriers only. We estimated 1) Z-scores per region (similar calculations as in^31^) and created 2) global index and 3) intraindividual standard deviation (similar calculations as in ^21^) as well as 4) regional intradeviation (RID) score.

1. *Z-scores*. Specifically, Z-scores for CNV carriers and non-carriers were calculated based on the mean and standard deviation from the non-carriers as shown in Eq. (1):
  (1) $$Z_{if}=\frac{\left(X_{if}-M_{if}\right)}{SD_{if}}$$
  Where Z_if_ is the standardized value for brain region *i* in feature *f* (i.e., cortical thickness, surface area, or subcortical volume), and X_if_ is the regional value for brain region *i* for feature *f*, M_if_ and SD_if_ represent the mean and standard deviation, respectively, for brain region *i* using feature *f* across the non-carriers. Thus, for every individual we obtained a vector of standardized Z-scores across 68 cortical regions for cortical thickness and cortical surface area, and 14 subcortical regions.
2. *Global index:* We created an individualized global index (GI) for cortical thickness, cortical surface area and subcortical volume, respectively, by calculating the mean Z-score across the cortical and subcortical regions as shown in Eq. (2)
  (2) $$GI_{f}=\frac{1}{n_{f}}\sum_{i=1}^{n_{f}}Z_{if}$$
  where GI_f_ is the global index for feature *f*, n is the total number of brain regions for feature *f*, and Z_if_ is the standardized value for the brain region *I* for feature *f* derived from Eq. (1).
3. *Intraindividual standard deviation:* Furthermore, we also calculated the intraindividual standard deviation (iSD) across the Z-scores for cortical thickness, cortical surface area, and subcortical volume to obtain measures of within-individual variability, as shown in Eq. (3):
  (3) $$iSD_{f}=\sqrt{\frac{\sum_{i=1}^{n_{f}}{\left(Z_{if}-GI_{f}\right)}^{2}}{n_{f}-1}}$$
  where the n_f_ is total number of brain regions for feature *f*, Z_if_ is the standardized value for brain region *i* for feature *f*, GI_f_ is the global index for feature *f* (i.e., mean Z-score across brain regions for an individual) as derived from Eq. (2). A low iSD indicates that an individual’s Zscores across brain regions are relatively consistent and do not vary much across brain regions, while a high iSD indicates that the Z-score across brain regions are relatively inconsistent, indexing a more variable brain.
4. *Regional intra-deviation score:* Finally, to identify regions that diverge more than expected from an individual’s global index and intraindividual standard deviation, we created a regional intra-deviation (RID) score calculated using Eq. (4) for every brain region across feature *f*:
  (4) $$RID_{f}=\frac{\left(Z_{if}-GI_{f}\right)}{iSD_{f}}$$
  where the Z_if_ is the standardized value for brain region *i* for feature *f* and GI_f_ is the global index for feature *f* as shown in Eq. (2.). The iSD_f_ reflects the standard deviation for the Z-score across brain regions in feature *f* as formulated in Eq. (3). Here, we define regions that are less affected as those that do not follow the global tendency in the data, whereas the regions that exceed the global tendency of the data are considered to be more affected. To establish brain-cognition relationships between the brain measures and cognition, we tested for associations between RID and Z-scores and cognitive ability (supplementary note 3, Figure S1, Table S1).

### Statistical analyses

All statistical analyses were conducted in R studio v4.0.0 and brain visualizations were created using the ENIGMA toolbox^32^. For the per-CNV analyses, we tested for group differences by including carrier status (i.e., either carrier or non-carrier) in a linear regression model. The deletion and duplication carriers were tested separately with their corresponding matched non-carrier group used as the reference. The estimated standardized beta values were extracted from the models and are presented in the results as a measure of effect size. The p-values underwent a False Discovery Rate (FDR)^33^ adjustment to account for multiple comparisons for each of the four CNV groups. Corrected p-values below .05 were considered statistically significant. Three main analyses were performed: First, in line with the conventional mass-univariate analysis approach, we performed group comparisons on the Z-scores across all the ROIs for cortical thickness, cortical surface area and subcortical volume (FDR corrected for 150 comparisons). Second, we compared the global index, and intraindividual standard deviation and mean corrected intraindividual standard deviation values between carriers and non-carriers (FDR corrected for 12 comparisons). The mean corrected intraindividual standard deviation represents the intraindividual standard deviation after regressing out the global index, as the mean values tend to be correlated with the standard deviation. Third, for the RID scores, group comparisons were computed between carriers and non-carriers for all ROIs for cortical thickness, cortical surface area, and subcortical volume (FDR corrected for 150 comparisons). Due to missing values in some brain regions, the analyses were restricted to individuals with complete observations for the feature that was analyzed (i.e., cortical thickness, cortical surface area, and subcortical volume). Sensitivity analyses were conducted for the significant RID score differences by adjusting for affection status (i.e., known psychiatric or neurological diagnoses). In addition, we examined the interaction term between carrier status and affection status and between carrier status and cognitive ability. Finally, we compared the brain profile of significant differences in RID scores to the significant differences in Z-scores adjusted for the global index.

## Results

### Global measures

The group differences in the global index and the intraindividual standard deviation measures are presented in Table 3 with reference values for the non-carrier groups in Table S2. The 1q21.1 distal deletion carriers had a lower global index for surface area, whereas the 15q11.2 BP1-BP2 deletion carriers had a lower global index for surface area and a higher global index for cortical thickness. In addition, the 15q11.2 BP1-BP2 duplication carriers had a lower global index for cortical thickness. Furthermore, there was a higher intraindividual standard deviation for cortical surface for both the 1q21.1 distal duplication carriers (both for the mean corrected and uncorrected measure) and the 15q11.2 BP1-BP2 deletion carriers (only for the mean corrected measure), as well as a higher intraindividual standard deviation for cortical thickness in the 15q11.2 BP1-BP2 deletion carriers (both for the mean corrected and uncorrected measure). With one exception, correlations between the intraindividual standard deviation measures across CNV groups did not show any significant differences (supplementary note 4, Figure S2).

### 1q21.1 distal copy number variant

**Figure 1:**
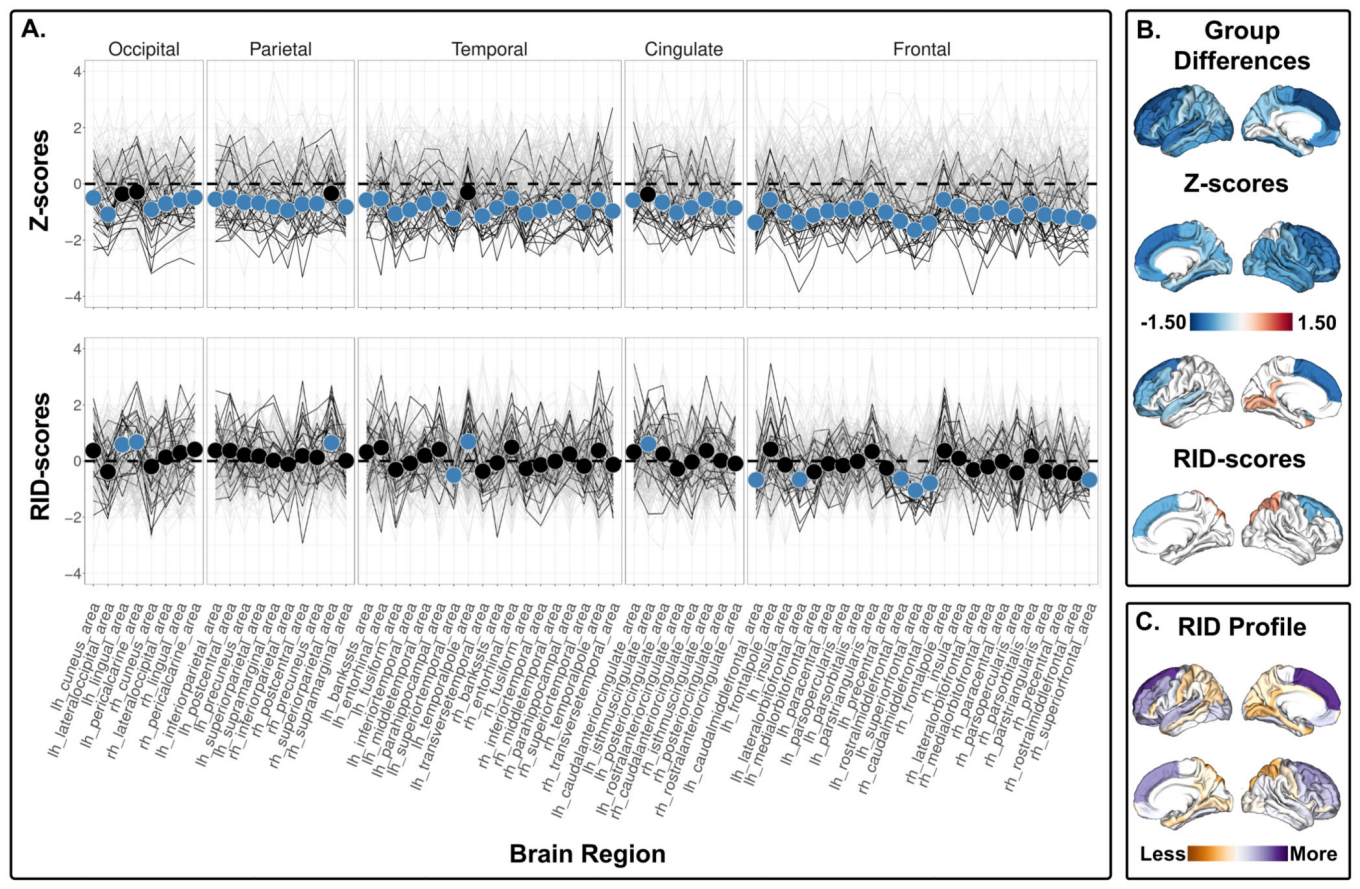
Cortical surface area comparison between 1q21.1 distal deletion carriers and non-carriers. A) Top panel shows z-scores - group differences in regional cortical surface area. Bottom panel shows RID-scores - group differences in regional cortical surface area that are scaled to the individual’s own global index. Non-carriers are represented by gray lines, and 1q21.1 distal deletion carriers are represented by black lines. Blue dots indicate significant differences. The insular cortex is included under frontal cortex for visualization purposes. B) Top panel displays the significant differences in Z-scores, and the bottom panel shows the significant differences in RID-scores. Blue-red diverging maps represent the effect size. C) Spatial distribution of all the mean differences in RID scores. Please note that all values are shown regardless of significance. Yellow-purple diverging maps represent the direction of the mean differences. Increased yellow intensity represents values that are less deviant than the overall global mean difference in cortical surface area, and increased purple intensity represents values that are more deviant than the overall global mean difference in cortical surface area. Z- and RID-scores are based on raw values adjusted for age, age^2^, sex, and intracranial volume on site harmonized data.

**Figure 2:**
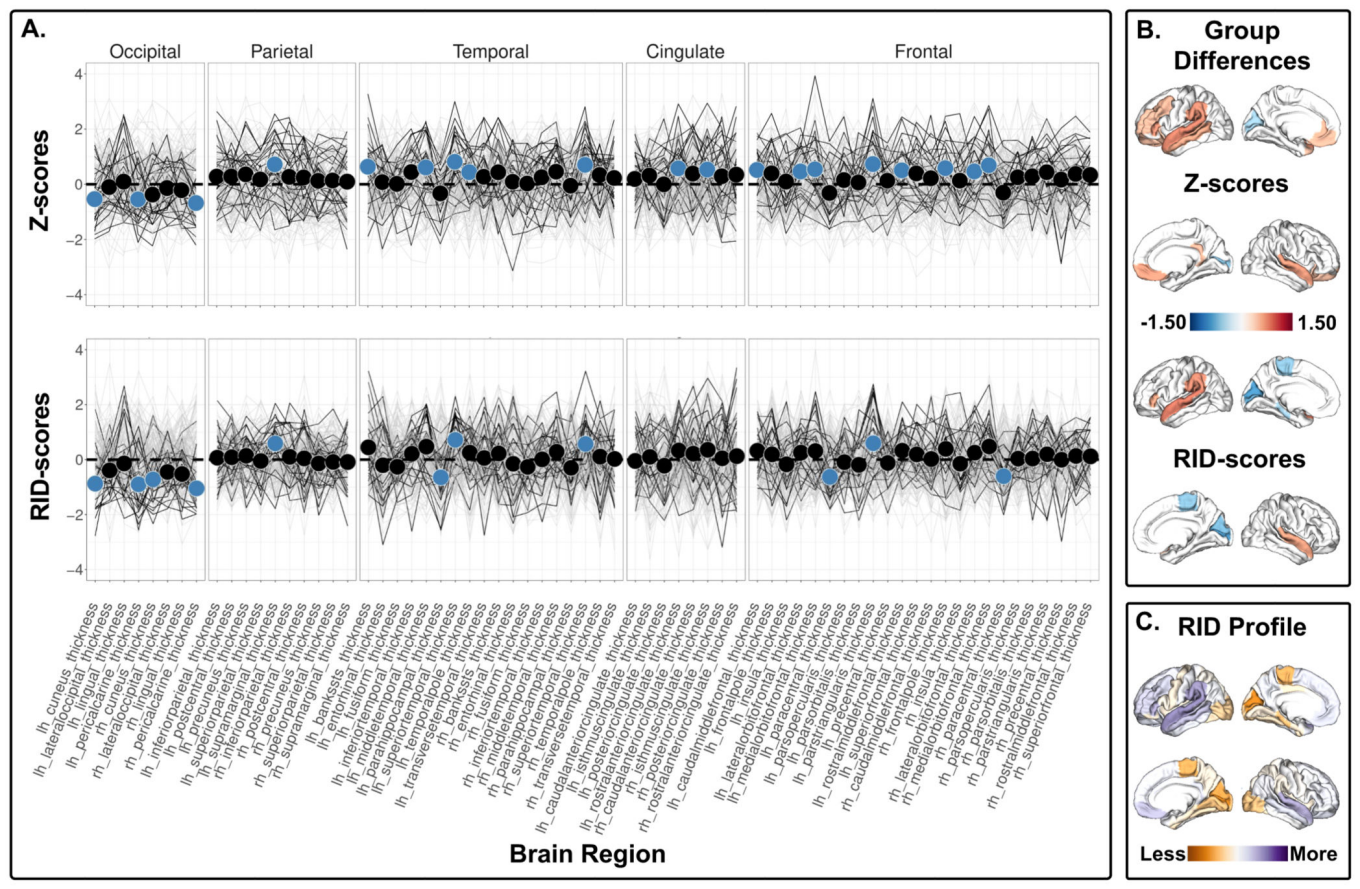
Cortical thickness comparison between 1q21.1 distal deletion carriers and non-carriers. A) Top panel shows z-scores - group differences in regional cortical thickness. Bottom panel shows RID-scores - group differences in regional cortical thickness that are scaled to the individual’s own global index. Non-carriers are represented by gray lines, and 1q21.1 distal deletion carriers are represented by black lines. Blue dots indicate significant differences. The insular cortex is included under frontal cortex for visualization purposes. B) Top panel displays the significant differences in Z-scores, and the bottom panel shows the significant differences in RID-scores. Blue-red diverging maps represent the effect size. C) Spatial distribution of all the mean differences in RID scores. Please note that all values are shown regardless of significance. Yellow-purple diverging maps represent the direction of the mean differences. Increased yellow intensity represents values that are less deviant than the overall global mean difference in cortical thickness, and increased purple intensity represents values that are more deviant than the overall global mean difference in cortical thickness. Z- and RID-scores are based on raw values adjusted for age, age^2^, sex, and intracranial volume on site harmonized data.

***The 1q21.1. distal deletion*** carriers showed widespread lower cortical surface area with significant differences in 63 ROIs using Z-scores (Figure 1a-b, top; Table S3), and exhibited a higher RID score for cortical surface area in regions within the occipital, superior parietal, temporal pole and posterior cingulate cortex, as well as lower RID scores in regions within the superior temporal and frontal regions (Figure 1a-c, bottom, Table S4). Further, 1q21.1. distal deletion carriers showed higher cortical thickness compared to non-carriers in 19 ROIs using Z-scores (Figure 2a-b, top, Table S3), in addition to lower RID scores for regions within the occipital lobe and paracentral lobule and higher RID scores for regions within the superior temporal and inferior frontal cortex (Figure 2a-c, bottom, Table S4). The 1q21.1 distal deletion carriers also exhibited lower subcortical volume in left thalamus and right nucleus accumbens (Table S3), and lower RID score for the left thalamus (Table S4). All the significant RID score differences survived adjustment for affection status. The interaction term between carrier status and affection status was not associated with the significant RID scores (supplementary note 5, Table S5). A subset of the significant RID scores were implicated in the brain-cognition RID map (Figure S1). However, we did not observe any significant interactions between carrier status and cognitive ability on any of the significant RID scores (supplementary note 6, Table S6). The results yielded more significant group differences in RID scores (i.e., 24) compared to Z-scores adjusted for the global index between 15q11.2 BP1-BP2 deletion carriers and non-carriers (i.e., 13, supplementary note 7, Figure S3, Table S7). ***The 1q21.1 distal duplication*** carriers showed higher cortical surface area in the right pars opercularis and right superior frontal gyrus, and lower volume in the right and left hippocampus compared to non-carriers (Table S8). Using RID scores, no significant differences in the ROIs were found (Table S9).

### 15q11.2 BP1-BP2 copy number variant

**Figure 3:**
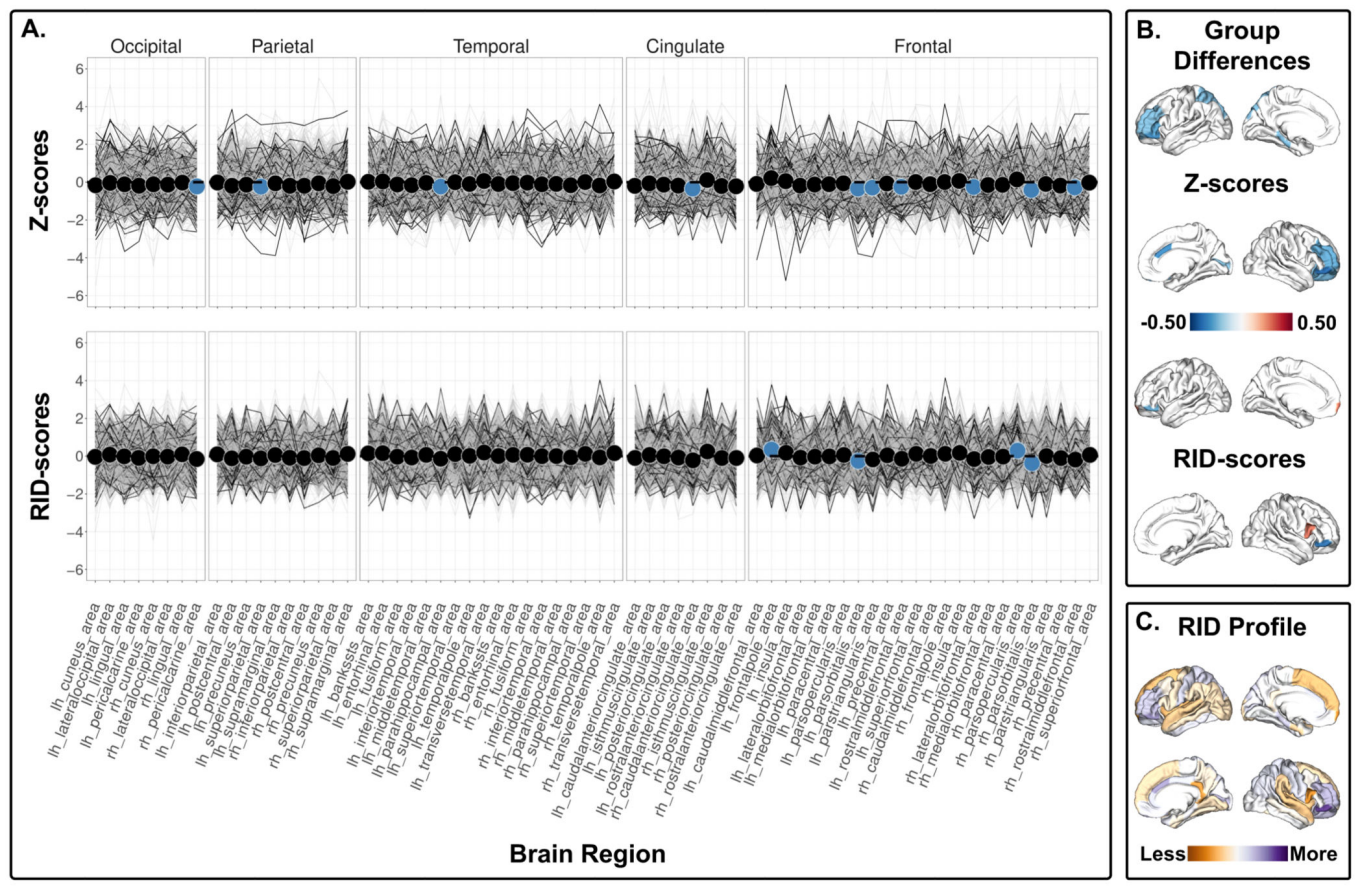
Cortical surface area comparison between 15q11.2 BP1-BP2 deletion carriers and non-carriers. A) Top panel shows z-scores - group differences in regional cortical surface area. Bottom panel shows RID-scores - group differences in regional cortical surface area that are scaled to the individual’s own global index. Non-carriers are represented by gray lines, and 15q11.2 BP1-BP2 deletion carriers are represented by black lines. Blue dots indicate significant differences. The insular cortex is included under frontal cortex for visualization purposes. B) Top panel displays the significant differences in Z-scores, and the bottom panel shows the significant differences in RID-scores. Blue-red diverging maps represent the effect size. C) Spatial distribution of all the mean differences in RID scores. Please note that all values are shown regardless of significance. Yellow-purple diverging maps represent the direction of the mean differences. Increased yellow intensity represents values that are less deviant than the overall global mean difference in cortical surface area, and increased purple intensity represents values that are more deviant than the overall global mean difference in cortical surface area. Z- and RID-scores are based on raw values adjusted for age, age^2^, sex, and intracranial volume on site harmonized data.

**Figure 4:**
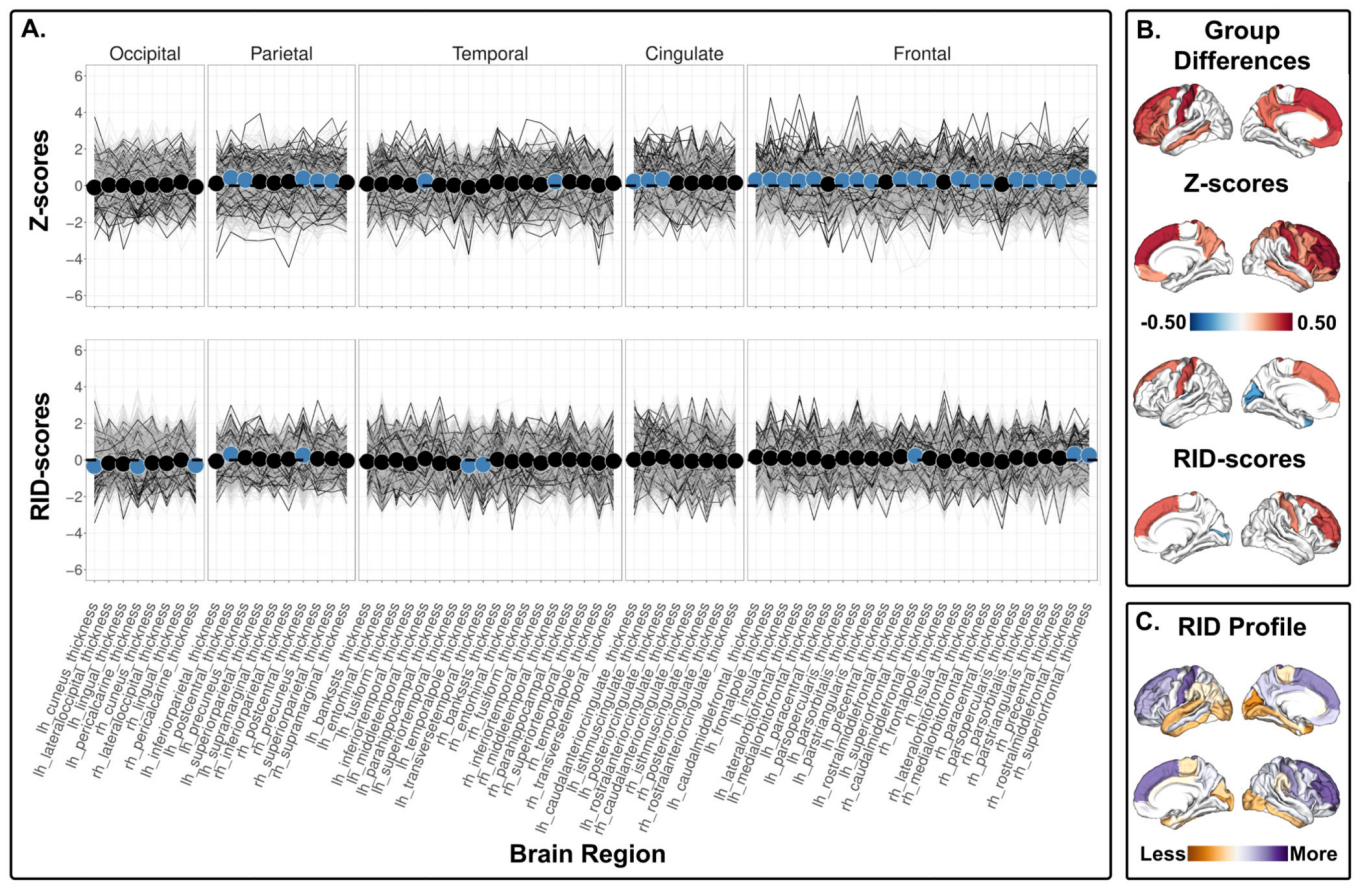
Cortical thickness comparison between 15q11.2 BP1-BP2 deletion carriers and non-carriers. A) Top panel shows z-scores - group differences in regional cortical thickness. Bottom panel shows RID-scores - group differences in regional cortical thickness that are scaled to the individual’s own global index. Non-carriers are represented by gray lines, and 15q11.2 BP1-BP2 deletion carriers are represented by black lines. Blue dots indicate significant differences. The insular cortex is included under frontal cortex for visualization purposes. B) Top panel displays the significant differences in Z-scores, and the bottom panel shows the significant differences in RID-scores. Blue-red diverging maps represent the effect size. C) Spatial distribution of all the mean differences in RID scores. Please note that all values are shown regardless of significance. Yellow-purple diverging maps represent the direction of the mean differences. Increased yellow intensity represents values that are less deviant than the overall global mean difference in cortical thickness, and increased purple intensity represents values that are more deviant than the overall global mean difference in cortical thickness. Z- and RID-scores are based on raw values adjusted for age, age^2^, sex, and intracranial volume on site harmonized data.

***The 15q11.2 BP1-BP2 deletion*** carriers showed lower cortical surface area in 10 ROIs using Z-scores (Figure 3a-b, top, Table S10), and higher RID scores for the left frontal pole and right pars opercularis surface area, but lower RID scores for the left and right pars orbitalis surface area compared to non-carriers (Figure 3a-c, bottom, Table S11). For cortical thickness, the 15q11.2 BP1-BP2 deletion carriers showed higher cortical thickness in 30 regions using Z-scores (Figure 4a-b, top, Table S10). The RID scores for cortical thickness were lower in regions within occipital and temporal regions, and higher in motor and frontal regions compared to non-carriers (Figure 4a-c, bottom, Table S11). The 15q11.2 BP1-BP2 deletion carriers also showed lower Z-scores for left caudate, right pallidum and right nucleus accumbens (Table S10). All significant RID scores remained significant after adjustment for affection status. No significant interactions between carrier status and affection status (Table S12, supplementary note 5) nor between carrier status and cognitive ability for the 15q11.2 BP1-BP2 deletion carriers were observed (Table S13, supplementary note 6). The results yielded more significant group differences in RID scores (i.e., 14) compared to Z-scores adjusted for global index (i.e., 12) between 15q11.2 BP1-BP2 deletion carriers and non-carriers (supplementary note 7, Figure S4, Table S14). ***The 15q11.2 BP1-BP2 duplication*** carriers showed lower cortical thickness in 11 ROIs and higher right superior frontal cortical surface area using Z-scores (Table S15) but showed no significant differences in the ROIs using RID-scores (Table S16).

## Discussion

The current study is the first to identify intraindividual variability differences in brain structure in CNV carriers. Using the intraindividual standard deviation measure, we observed higher variability in the regional effects for cortical surface area in both 1q21.1 distal duplication and 15q11.2 BP1-BP2 deletion carriers, and higher variability in the regional effects for cortical thickness for the 15q11.2 BP1-BP2 deletion carriers, compared to non-carriers. Using RID scores, we find that a subset of brain regions diverged significantly from non-carriers for both the 1q21.1 distal and 15q11.2 BP1-BP2 deletion carriers. We also find a higher number of significant regional differences using RID scores compared to the conventional global covariation approach. The current results hold promise for identifying specific CNV-associated brain profiles by targeting regional differences using an individualized approach, which are overlooked in studies applying conventional brain MRI measures.

In line with previous results^15^, the 1q21.1 distal deletion carriers showed lower global cortical surface area compared to non-carriers. The observed differences in Z-scores indicate widespread lower cortical surface area, whereas the RID scores indicate that the cortical surface area in posterior and primary sensory regions (i.e., lingual, pericalcarine, superior parietal, isthmus of the cingulate gyrus) are less affected and frontal and association cortices (i.e., caudal middle frontal, lateral orbitofrontal, rostral middle frontal, superior frontal cortex) are more affected. Thus, the observed regional Z-score group differences along lateral and medial parietal to lateral inferior temporal and motor cortex appear to be largely reflective of the global effect. A subset of the significant RID scores (i.e., the superior temporal gyri and left supramarginal gyrus cortical thickness and left lateral orbitofrontal and left lateral superior temporal gyrus cortical surface area) was associated with cognitive ability in non-carriers. However, the effect sizes are low, and the current sample size of CNV carriers is too small to reliably detect such brain-cognition associations.

The 15q11.2 BP1-BP2 deletion showed a higher global cortical thickness compared to non-carriers, primarily concentrated in the frontal cortex, recapitulating previously reported group differences in cortical thickness^16^. We complement these findings by showing group differences in RID scores, which indicates that the cortical thickness in sensory cortices (i.e., cuneus and pericalcarine area) are less affected, and the association cortices (i.e., rostral middle frontal and superior frontal cortex) are more affected by the deletion. The association cortices that show cortical thickness differences using RID scores are regions that underlies complex cognitive functions^23–25^, and may subserve the lower cognitive performance in 15q11.2 BP1-BP2 deletion carriers compared to controls^14,34^.

Notably, some findings deviate from the interpretation of a less affected sensorimotor cortex and a more affected association cortex. Both the 1q21.1 distal and 15q11.2 BP1-BP2 deletion carriers show evidence for a relatively less affected cortical surface area and cortical thickness, respectively, in the left temporal pole. We also find that the cortical thickness of the postcentral gyri, a primary somatosensory region, is more affected in the 15q11.2 BP1-BP2 deletion carriers. To speculate, this may be associated with the motor delay observed in clinically affected 15q11.2 BP1-BP2 deletion carriers^35^. For cortical surface area in the 15q11.2 BP1-BP2 deletion carriers, we find inconsistent effects for frontal regions: although we observe a relatively more different bilateral pars orbitalis, we also find evidence for a less different left frontal pole and right pars opercularis. Furthermore, we did not find significant differences in RID scores in the 15q11.2 BP1-BP2 duplication carriers, nor in the 1q21.1 distal duplication carriers. The results complement previous findings of lower effect sizes in brain measures for duplication versus deletion carriers^6,7^, and thus may support that deletion carriers distort the anatomical relationships in the brain more than duplication carriers.

Global and frontal regional group differences in cortical thickness are prominent brain features of several neurodevelopmental disorders, including autism spectrum disorder^36^ and schizophrenia^37^. Thus, group differences in brain structure may be confounded by individuals with neurodevelopmental or psychiatric disorders. Here, all the significant RID score differences in 1q21.1 distal and 15q11.2 BP1-BP2 deletions survived adjustment for affection status, and there were no interaction effects between carrier status and affection status on the significant RID scores.

The current results implicate novel mechanisms in neurodevelopment. Compelling candidates for the changes in the 1q21.1 distal CNV are the human specific *NOTCH2NL* genes, which have been linked to the evolutionary expansion of the human neocortex^38,39^. NOTCH signaling is important for outer radial glia cell self-renewal, which are thought to contribute to cortical expansion^40^. Deletion of the *NOTCH2NL* genes in human cortical organoids yields smaller organoids compared to controls^38^ and *NOTCH2NL* increases the number of cycling basal progenitors in the mouse embryonic neocortex^41^. Thus, *NOTCH2NL* could yield a potential mechanistic link between the assumed lower gene expression levels in 1q21.1 distal deletion carriers and the lower cortical surface area, possibly important for the expansion of frontal regions.

Among the four genes in the 15q11.2 BP1-BP2 loci^42^, *CYFIP1* has gained considerable interest due to its association to schizophrenia^43,44^ and autism^45–47^. *CYFIP1* exhibits high expression levels in the developing mouse brain^47^. *CYFIP1* has also been linked to variation in cortical surface area^48^, as well as various cellular phenotypes, including myelination^49^, neurite length and branch number, cell size^50^, dendritic spine formation^51^ and regulation of radial glia cells^52^. Notably, *CYFIP1* haploinsufficiency lower myelination thickness in rats^49^. Cortical thickness, as estimated with MRI, has been suggested to be influenced by myelination^53^. Thus, the higher cortical thickness observed in 15q11.2 BP1-BP2 deletion carriers may be due to altered myelination in the brain, possibly with somatosensory cortex being particularly sensitive to these alterations. *CYFIP1* deficiency has also been associated with functional connectivity deficits in motor cortices, as well as aberrant motor coordination in mice^54^. Finally, it should be noted that the 1q21.1 distal and the 15q11.2 BP1-BP2 loci span several genes, and genes within CNVs are likely to be involved in multifaceted genetic interactions^55^. More research is needed to identify the causative biological mechanisms of the brain structural phenotypes.

This study has strengths and limitations. We use an intraindividual variability approach to examine brain metrics that are related to an individual’s own inter-regional brain profile. By examining metrics that consider the variation within individuals, it is possible to map the heterogeneity and deviations in CNV carriers compared to non-carriers. However, variability measures should be interpreted with caution, as some effects on the brain may be so extreme that further deviations are unlikely to be observed. That is, CNVs may yield large effects on brain structure, but only to a certain extent due to biological constraints. Thus, we urge caution when interpreting intraindividual standard deviation in brain measures as ceiling and floor effects may bias the variability metrics. Still, we identify structures that are significantly less different or more different relative to the mean difference, indicating sufficient variability in the individualized brain metrics. About 1/2 (1q21.1 distal) and 2/3 (15q11.2 BP1-BP2) of the carriers are derived from the UK Biobank, which has a healthy volunteer bias^56^, possibly yielding underestimations of brain structural differences. However, this is somewhat counter-balanced by the ENIGMA-CNV dataset that is likely to increase the heterogeneity in the study sample (although some datasets are likely to have similar bias towards healthy individuals as the UK Biobank). Indeed, the variability observed in brain structure within individuals underscores the heterogeneity between and within individuals in the sample. Future studies with larger sample sizes are needed to examine the phenotypic heterogeneity observed in CNV carriers.

The results of the current study aid our understanding of 1q21.1 distal and 15q11.2 BP1-BP2 CNV brain profiles by identifying regional differences using intraindividual variability metrics, which has the potential to give better insight into the neuronal mechanisms in neurodevelopment and risk for psychiatric diseases. We find evidence for regional differences beyond the global differences in brain structure, where the spatial effects partly support the hypothesis of less affected sensorimotor cortex and more affected association cortex in both the 1q21.1 distal and 15q11.2 BP1-BP2 deletion carriers.

## Supplementary Material

Supplementary Material
