# Supplementary material for: Beyond the Global Brain Differences: Intra-individual Variability Differences in 1q21.1 Distal and 15q11.2 BP1-BP2 Deletion Carriers
Source: Biol Psychiatry. Author manuscript; Available in PMC 2024 Jan 1. (PMC7615370; doi:10.1016/j.biopsych.2023.08.018)
Supplement: Supplementary Material [file EMS190337-supplement-Supplementary_Material.zip › 1-s2.0-S0006322323015305-mmc1.pdf]

SUPPLEMENTARY INFORMATION

Beyond the Global Brain Differences: Intra-individual Variability Differences in 1q21.1 Distal and 15q11.2 BP1-BP2 Deletion Carriers

Boen *et al.*

Content in this file:

Supplementary note 1: Sampling procedure

Supplementary note 2: MRI quality control

Supplementary note 3. Association between RID scores and Z-scores and cognitive ability.

Supplementary note 4: Associations between the intraindividual standard deviation values for cortical surface area, cortical thickness, and subcortical volume.

Supplementary note 5. RID scores and affection status

Supplementary note 6. RID scores and cognitive ability in 1q21.1 distal deletion and 15q11.2 BP1-BP2 deletion carriers.

Supplementary note 7. Comparison between the conventional case-control analysis adjusted for the global index and the RID score approach.

Supplementary references

Content available in separate Excel file:

Supplementary Appendix 1: Technical details of the scanners and acquisition parameters used at the participating sites.

Table S1. Associations between cognitive ability and Z-scores and RID-scores in the UK Biobank.

Table S2. Reference values for the global index and intraindividual standard deviation

Table S3. Group differences in Z-scores for 1q21.1 distal deletion carriers.

Table S4. Group differences in RID-scores for 1q21.1 distal deletion carriers.

Table S5. Carrier status (i.e., 1q21.1 distal deletion vs non-carriers) and affection status on RID-scores.

Table S6. Carrier status (i.e., 1q21.1 distal deletion vs non-carriers) and cognitive ability on RID-scores.

Table S7. Group differences in Z-scores for 1q21.1 distal deletion carriers, adjusted for the global index.

Table S8. Group differences in Z-scores for 1q21.1 distal duplication carriers.

Table S9. Group differences in RID-scores for 1q21.1 distal duplication carriers.

Table S10. Group differences in Z-scores for 15q11.2 BP1-BP2 deletion carriers.

Table S11. Group differences in RID-scores for 15q11.2 BP1-BP2 deletion carriers.

Table S12. Carrier status (i.e., 15q11.2 BP1-BP2 deletion vs non-carriers) and affection status on RID-scores.

Table S13. Carrier status (i.e., 15q11.2 BP1-BP2 deletion vs non-carriers) and cognitive ability on RID-scores.

Table S14. Group differences in Z-scores for 15q11.2 BP1-BP2 deletion carriers, adjusted for the global index.

Table S15. Group differences in Z-scores for 15q11.2 BP1-BP2 duplication carriers.

Table S16. Group differences in RID-scores for 15q11.2 BP1-BP2 duplication carriers.

Supplementary note 1: Sampling procedure

The included samples were drawn from a pool of participants from the ENIGMA-CNV working group and the UK Biobank. Participants flagged with either a 1q21.1 distal deletion, 1q21.1 distal duplication, 15q11.2 BP1-BP2 deletion or 15q11.2 BP1-BP2 duplication were included in the study. For the participants pooled from the ENIGMA-CNV consortium, identification of the CNV carriers was derived as described in previously published articles on the 1q21.1 distal and 15q11.2 BP1-BP2. For the UK Biobank sample, we identified CNVs based on the returned dataset from Crawford et al.(1). All participants flagged to have a CNV as previously reported in Crawford et al. were removed from downstream analyses, except for those flagged with the 1q21.1 distal or the 15q11.2 BP1-BP2 CNVs. A matched non-carrier group for each of the four CNV samples were extracted using the MatchIt function in R(2). The non-carrier group was matched to each carrier on age, sex, scanner site and ICV. The sample size of the non-carrier group is based on previous published studies. For the 1q21.1 distal deletion, sensitivity analyses indicated that group differences can be detected using 1:1 ratio sample comparison due to large effect sizes(3). For the 15q11.2 BP1-BP2 deletion, previous power analysis has found a 1:4 ratio sample comparison suitable detect small to medium effect sizes(4). Here, we used a matched non-carrier group that was five times larger than the carrier group as we expected some reduction in sample size due to missing data (see below). Furthermore, we also harmonized the data across scanner sites and use a mega-analytical approach to test for group differences, which has been reported to yield increased power compared to studies using a meta-analytical approach with scanner site included as a covariate(5).

Supplementary note 2: MRI quality control

The MRI data from the UK Biobank underwent an automatic quality control procedure, using the derived Euler numbers to exclude participants with Euler numbers that dropped below four standard deviations from the rest of the sample. For the ENIGMA-CNV sample, all cohorts were asked to complete the cortical quality control that includes visual and statistical evaluation of the included participants (https://enigma.ini.usc.edu/protocols/imaging-protocols/). Further, after matching each carrier to five non-carriers, we examined the distribution of the Z-score in each of the four derived samples. Here, non-carriers that exhibited a highly atypical Z-score distribution (i.e., severe change in a regional Z-score within participants, i.e, > 6SD) were removed from the sample. The non-carriers were removed from the sampling pool, and we reran the matching procedure to obtain five non-carriers per carrier. We detected one regional value with a severe change in Z-score (i.e., > 7SD) among one 15q11.2 BP1-BP2 deletion carrier from the UK Biobank. This region was excluded for this participant (i.e., regional value was set to NA) in downstream analyses. As the statistical procedure used in the current study is sensitive to individuals that do not have a full observation across the feature of interest (i.e., cortical thickness, surface area, and subcortical volume), we only included individuals with full observations for the feature of interest. Thus, the number of individuals included in the analyses differs between features. The number of individuals included in the analyses is included in the supplementary tables. The four samples were derived from a total of 61 scanner sites (including scanner sites from the UK Biobank). Thus, to account for systematic differences between scanner sites, we ran each of the four subsamples through ComBat, which is an instrument for data harmonization that increases the statistical power and can harmonize data with missing values(5).

Supplementary note 3. Association between RID scores and Z-scores and cognitive ability.

To test the associations between the RID-scores and Z-scores and cognitive ability, we utilized data from the UK Biobank.

First, we created RID scores across all participants without a pathogenic CNV and with available imaging data (n = 40,312) using the sample's own mean and standard deviation to generate the Z-scores.

Second, we extracted cognitive scores from four different cognitive tasks that were available from the imaging visit (i.e., Data-Field 20016: fluid intelligence test (verbal and numeric reasoning), Data-Field 20023: reaction time test (simple processing speed), Data-Field 4282: digit span test (numeric working memory) and Data-Field 399: pairs matching test (episodic memory)). The intelligence measure is based on the number of correct responses on 13 questions, reaction time is based on the log transformed mean reaction time after the removal of trials with responses <50 ms and >2000 ms, digit span is based on the maximum number of digits remembered, and pairs matching is based on errors in the second round using a log+1 transformation. Further, we imputed values for the missing data using multivariate imputation by chained equations with classification and regression trees(6).

Third, we ran a principal component analysis across the test scores to get the first principal component across the four tasks, which we used as a measure of cognitive ability(7,8). We transformed the first principal component, such that higher values indicate better cognitive performance. The first principal component was highly correlated with number of correct responses on the intelligence task ($r = .985$, $p < .001$) and maximum number of digits remembered in the digit span task ($r = .506$, $p < .001$). Moreover, higher values of the first principal component were associated with fewer errors in the pairs matching task ($r = -.175$, $p < .001$) and faster reaction time in the reaction time test ($r = -.165$, $p < .001$).

Fourth, the data were divided into two samples, 75% of the participants went into a discovery sample, and the remaining 25% of the participants went into a replication sample, yielding two groups consisting of 30,234 and 10,078 participants, respectively. We created a linear regression model, where the measure of cognitive ability was included as the independent variable and the RID scores included as the dependent variable. This resulted in 150 comparisons. Here, we report the brain regions that were significant in the discovery sample after FDR correction (i.e., $P_{FDR} < .05$) across the 150 comparisons and were below the uncorrected $p < .05$ threshold in the replication sample (Table S1, left). The values were scaled to get a standardized beta value, serving as a measure of effect size. To compare the associations and effect sizes to conventional brain-cognition analyses, we redid the analysis using Z-scores (Table S1, right). The results are visualized in Figure S1. Interestingly, a subset of the RID scores that differed significantly between 1q21.1 distal deletion and non-carriers (i.e., left and right superior temporal gyri and left supramarginal gyrus cortical thickness and left lateral orbitofrontal and left lateral superior temporal gyrus cortical surface area) also displayed significant associations with cognitive ability in the UK Biobank non-carrier sample.

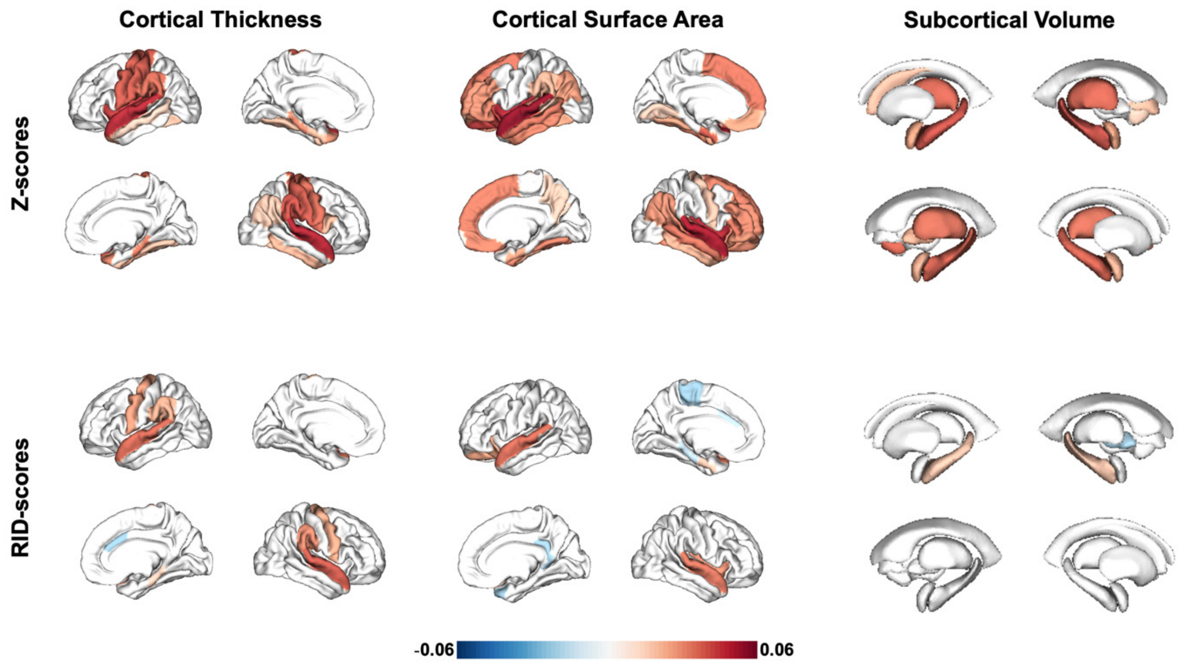

Figure S1. Associations between brain Z-scores (top) and RID scores (bottom) and cognitive ability measure [as estimated by the first principal component across the four tasks: Intelligence, reaction time, digit span and match pairing]. Blue-red diverging maps represent the effect size as derived from the discovery sample.

Supplementary note 4: Associations between the intraindividual standard deviation values for cortical surface area, cortical thickness, and subcortical volume.

To examine the associations between the intraindividual standard deviation values across the MRI-derived features, we correlated the iSD measures for cortical surface area, cortical thickness, and subcortical volumes for both CNVs. To increase statistical power for the non-carriers, we merged the two non-carrier groups in the 1q21.1 distal and 15q11.2 BP1-BP2 CNV groups, respectively. To test if the correlations between deletions and duplication carriers statistically differ from each other, we used the Fisher's Z test in the cocor package in R(9). The associations across CNV carriers and non-carriers are visualized in Figure S2.

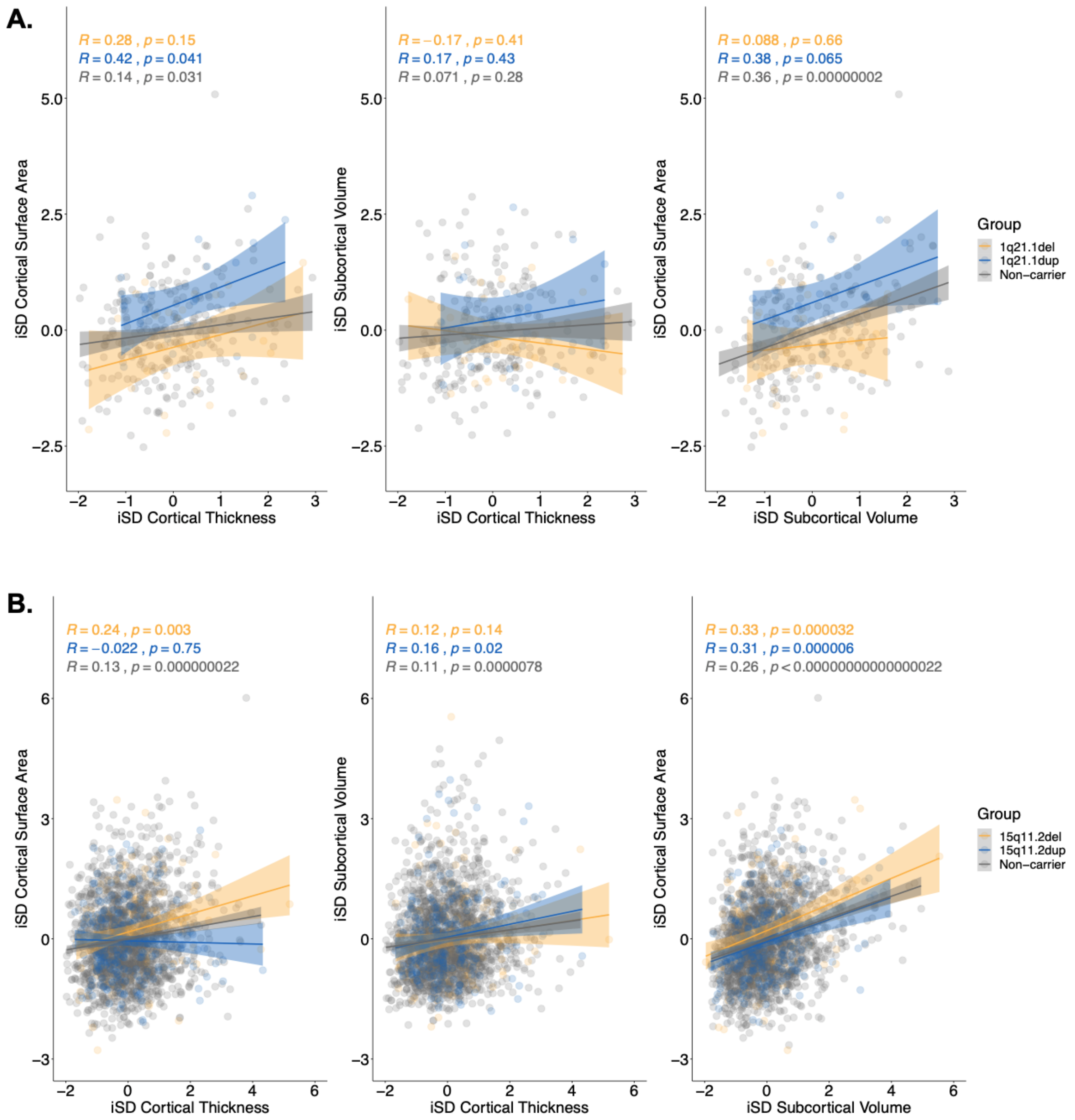

Figure S2. Correlations between intraindividual standard deviation values (iSDs) for cortical thickness, surface area, and subcortical volume. A) Correlations across 1q21.1 distal deletion and duplication carriers and non-carriers. B) Correlations across 15q11.2 BP1-BP2 deletion and duplication carriers and non-carriers. Correlation coefficients and p-values for each group are presented in the figures and are matched to the color of each group. Yellow = deletion carriers, blue = duplication carrier, grey = non-carrier. iSD = intraindividual standard deviation.

For the 1q21.1 distal deletion and duplication, we did not find evidence for a statistical difference in the correlations between iSD cortical thickness and iSD cortical surface area ($Z = -0.934$, $p = 0.350$), iSD cortical thickness and iSD subcortical volume ($Z = 0.962$, $p = 0.336$), or iSD subcortical volume and iSD surface area ($Z = -1.053$, $p = 0.292$).

For the 15q11.2 BP1-BP2 deletion and duplication, the correlation between iSD cortical thickness and iSD surface area were significantly different from each other ($Z = 3.207$, $p = 0.001$, $P_{FDR} = 0.004$), whereas there was no statistical difference between the correlations of iSD cortical thickness and iSD subcortical volume ($Z = 0.477$, $p = 0.633$) or iSD cortical surface area and iSD subcortical volume ($Z = 0.271$, $p = 0.786$).

The overall pattern of the results indicates that a more variable brain, as indicated by regional variability across ROIs, tend to be a feature of the whole brain, as indicated by the correlations between the MRI-derived measures (i.e., cortical surface area, cortical thickness, subcortical volume). This is supported by the consistent positive correlations between the MRI-derived measures from the well-powered non-carrier groups. However, for the 15q11.2 BP1-BP2 CNV, this pattern appears to be slightly different, as the 15q11.2 BP1-BP2 duplication does not show evidence for an association between the regional variability across ROIs using cortical surface area and regional variability across ROIs using cortical thickness, which significantly differed from the pattern observed in the 15q11.2 BP1-BP2 deletion.

Supplementary note 5. RID scores and affection status

To further examine the significant RID results between the 1q21.1 distal deletion carriers and non-carriers and 15q11.2 BP1-BP2 deletion carriers, we ran additional analyses to test the effect of affection status on the significant RID scores. Here, we included 1) affection status (i.e., having a known psychiatric or neurological diagnosis, or an F or G-ICD diagnosis for the UK Biobank participants, coded as 0 (none) and 1 (dx)) as a covariate and 2) an interaction term between affection status and copy number. The affection status was distributed as follows: 10 (33.3%) 1q21.1 distal deletion carriers and 18 (12%) non-carriers, and 19 (11.2%) 15q11.2 BP1-BP2 deletion carriers and 91 (10.7%) non-carriers.

All significant RID scores survived the adjustment for affection status for both the 1q21.1 distal deletion (Table S5, top) and the 15q11.2 BP1-BP2 deletion (Table S12, top). None of the RID scores showed an interaction effect between copy number status (i.e., deletion or non-carrier, non-carrier group used as the reference) and affection status (affection status 0 (none) used as the reference) for either the 1q21.1 distal deletion (Table S5, top) or the 15q11.2 BP1-BP2 deletion (Table S12, bottom).

Supplementary note 6. RID scores and cognitive ability in 1q21.1 distal deletion and 15q11.2 BP1-BP2 deletion carriers.

The low effect sizes for the brain-cognition relationship described in supplementary note 3, indicate that brain-cognition associations are underpowered for the CNV groups. Nevertheless, to explore the possibility that the brain-cognition relationships are stronger in the 1q21.1 distal deletion and 15q11.2 BP1-BP2 deletion carriers compared to the non-carriers, we tested for a significant interaction effect between our calculated measure of cognitive ability and carrier status using linear regression. A significant interaction term would indicate that the brain-cognition relationship is stronger for one of the groups. For each of the 1q21.1 distal deletion and 15q11.2 BP1-BP2 deletion samples, including their corresponding matched non-carrier group, we followed the same approach for imputation and principal component procedure as outlined in supplementary note 3. The cognitive ability measure was lower for the 1q21.1 distal deletion carriers (Estimate = -.667, S.E. = .307, t-value = -2.169, $p = .033$) and the 15q11.2 BP1-BP2 deletion carriers (Estimate = -.392, S.E. = .097, t-value = -4.030, $p < .001$) compared to non-carriers. However, we did not find evidence for an interaction effect between copy number and cognitive ability on the RID scores for either the 1q21.1 distal deletion (Table S6) or the 15q11.2 BP1-BP2 deletion carriers (Table S13). These results - the effect sizes between RID scores and the cognitive ability measure using the full UK Biobank sample (standardized beta values > .06) and the lack of interaction term between carrier status and cognitive ability - indicate that the 1q21.1 distal deletion and 15q11.2 BP1-BP2 deletion samples are likely underpowered to detect reliable brain-cognition relationships.

Supplementary note 7. Comparison between the conventional case-control analysis adjusted for the global index and the RID score approach.

To compare the significant RID scores to the conventional case-control analyses, adjusted for the global index, we created a linear regression model using copy number status as the independent variable with the global index as covariate and Z-scores as the dependent variable. The global index for cortical surface area, cortical thickness, and subcortical volume was included for cortical surface area, cortical thickness, and subcortical volume Z-scores, respectively. The continuous values were scaled and P-values that were below .05 after FDR correction were considered significant.

The Z-score results and RID score results are visualized in Figure S3 for the 1q21.1 distal deletion and in Figure S4 for the 15q11.2 BP1-BP2 deletion. The results indicate that the regions with the largest effect sizes remained significant after adjusting for the global index, but also showed some non-overlapping brain regions with their corresponding RID profile. That is, for the adjusted Z-scores the right paracentral cortical surface area was also significantly different between the 1q21.1 distal deletion and non-carriers, and the lateral occipital cortical thickness, right caudal anterior cingulate cortical surface area, and right nucleus accumbens subcortical volume differed significantly between the 15q11.2 BP1-BP2 deletion and non-carriers.

There are some key differences in the methodological approach between the estimations of RID scores and mean correcting for the global effect using linear regression. Of note, in contrast to the RID score approach, which use the estimated global index for each individual as the reference, the global covariation approach utilizes the linear relationship between the global index and the regional Z-scores across the sample to adjust for the global index. Here, we observe that the standardized beta values were smaller for the adjusted Z-scores and yielded a lower number of significant ROIs compared to the group differences using RID

scores.

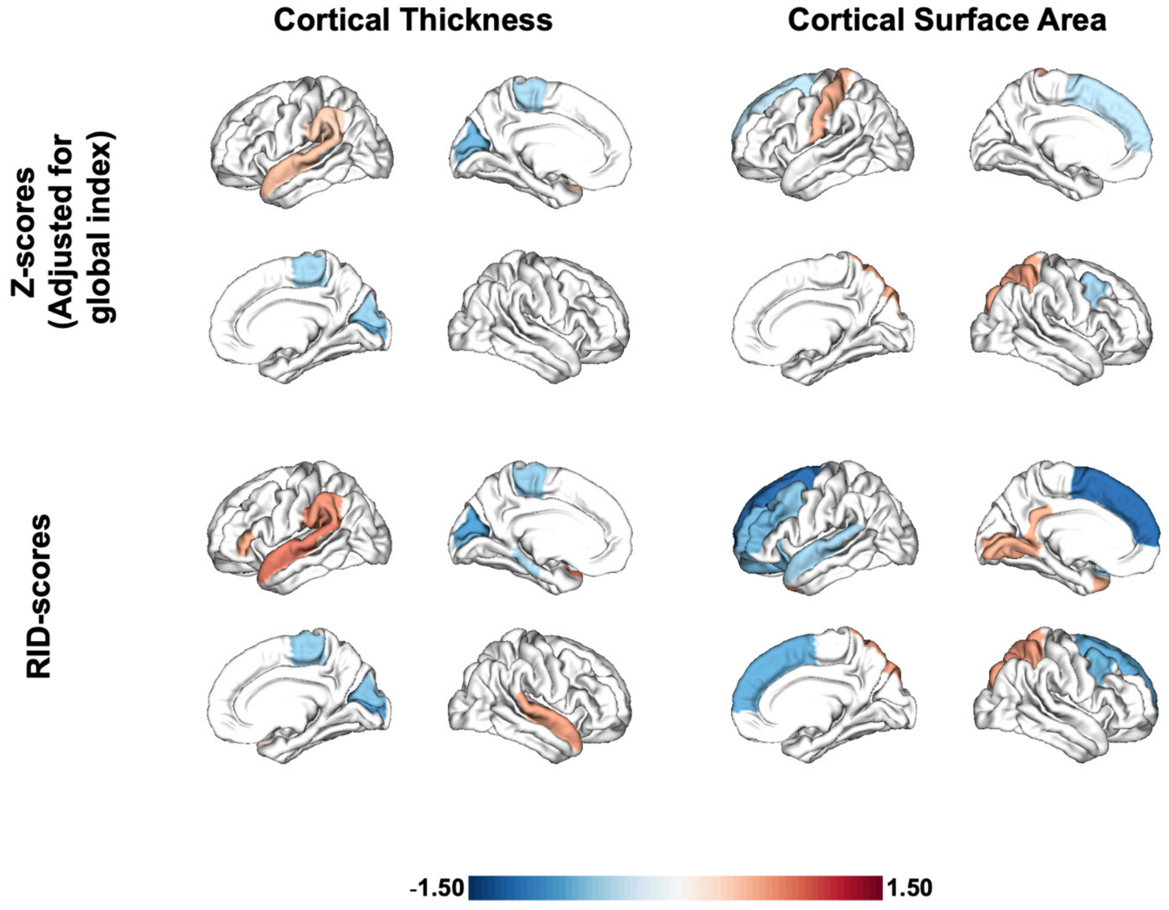

Figure S3. Comparison between the brain profile derived from adjusted Z-scores (top) and RID scores (bottom) derived from significant group differences between 1q21.1 distal deletion and non-carriers. Blue-red diverging maps represent the effect size.

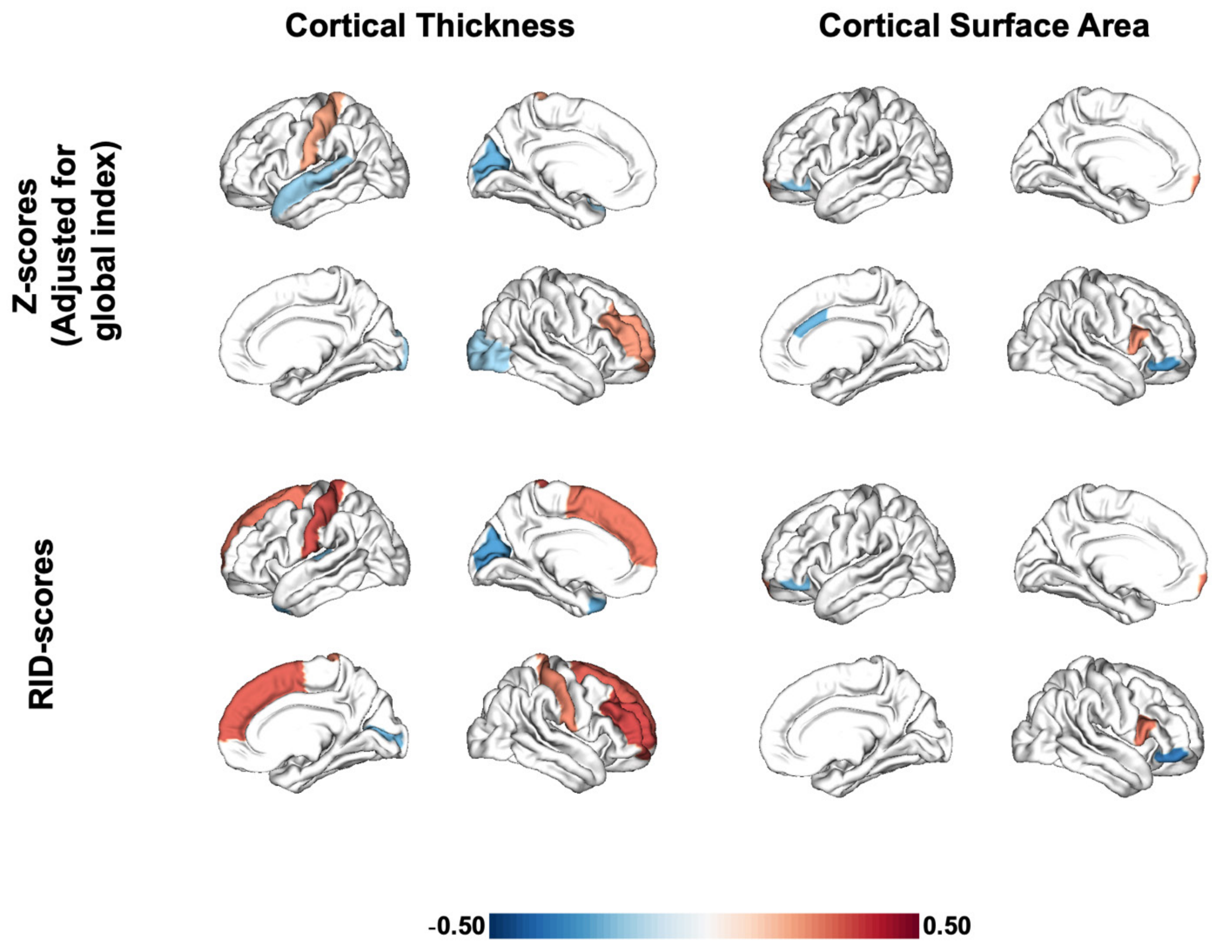

Figure S4. Comparison between the brain profile derived from adjusted Z-scores (top) and RID scores (bottom) derived from significant group differences between 15q11.2 BP1-BP2 deletion and non-carriers. Blue-red diverging maps represent the effect size.

Supplementary References

1. Crawford K, Bracher-Smith M, Owen D, Kendall KM, Rees E, Pardiñas AF, et al. Medical consequences of pathogenic CNVs in adults: analysis of the UK Biobank. J Med Genet. 2019 Mar 1;56(3):131–8.

2. Ho D, Imai K, King G, Stuart EA. MatchIt: Nonparametric Preprocessing for Parametric Causal Inference. J Stat Softw. 2011 Jun 14;42(1):1–28.

3. Sønderby IE, van der Meer D, Moreau C, Kaufmann T, Walters GB, Ellegaard M, et al. 1q21.1 distal copy number variants are associated with cerebral and cognitive alterations in humans. Transl Psychiatry. 2021 Mar 22;11(1):1–16.

4. Boen R, Kaufmann T, Frei O, van der Meer D, Djurovic S, Andreassen OA, et al. No signs of neurodegenerative effects in 15q11.2 BP1-BP2 copy number variant carriers in the UK Biobank. Transl Psychiatry. 2023 Feb 18;13(1):1–6.

5. Radua J, Vieta E, Shinohara R, Kochunov P, Quidé Y, Green MJ, et al. Increased power by harmonizing structural MRI site differences with the ComBat batch adjustment method in ENIGMA. NeuroImage. 2020 Sep 1;218:116956.

6. Buuren S van, Groothuis-Oudshoorn K. mice: Multivariate Imputation by Chained Equations in R. J Stat Softw. 2011 Dec 12;45:1–67.

7. Hepsomali P, Groeger JA. Diet and general cognitive ability in the UK Biobank dataset. Sci Rep. 2021 Jun 3;11(1):11786.

8. Fawns-Ritchie C, Deary IJ. Reliability and validity of the UK Biobank cognitive tests. PLOS ONE. 2020 Apr 20;15(4):e0231627.

9. Diedenhofen B, Musch J. cocor: A Comprehensive Solution for the Statistical Comparison of Correlations. PLOS ONE. 2015 Apr 2;10(4):e0121945.
